# Heterozygous Mutation in IκBNS Leads to Reduced Levels of Natural IgM Antibodies and Impaired Responses to T-Independent Type 2 Antigens

**DOI:** 10.3389/fimmu.2016.00065

**Published:** 2016-03-01

**Authors:** Gabriel K. Pedersen, Monika Ádori, Julian M. Stark, Sharesta Khoenkhoen, Carrie Arnold, Bruce Beutler, Gunilla B. Karlsson Hedestam

**Affiliations:** ^1^Department of Microbiology, Tumor and Cell Biology, Karolinska Institutet, Stockholm, Sweden; ^2^Department of Genetics, The Scripps Research Institute, La Jolla, CA, USA; ^3^Center for the Genetics of Host Defense, University of Texas Southwestern Medical Center, Dallas, TX, USA

**Keywords:** B-1 cells, transitional B cells, *nfkbid*, IκBNS, NF-κB

## Abstract

Mice deficient in central components of classical NF-κB signaling have low levels of circulating natural IgM antibodies and fail to respond to immunization with T-independent type 2 (TI-2) antigens. A plausible explanation for these defects is the severely reduced numbers of B-1 and marginal zone B (MZB) cells in such mice. By using an ethyl-*N*-nitrosourea mutagenesis screen, we identified a role for the atypical IκB protein IκBNS in humoral immunity. IκBNS-deficient mice lack B-1 cells and have severely reduced numbers of MZB cells, and thus resemble several other strains with defects in classical NF-κB signaling. We analyzed mice heterozygous for the identified IκBNS mutation and demonstrate that these mice have an intermediary phenotype in terms of levels of circulating IgM antibodies and responses to TI-2 antigens. However, in contrast to mice that are homozygous for the IκBNS mutation, the heterozygous mice had normal frequencies of B-1 and MZB cells. These results suggest that there is a requirement for IκBNS expression from two functional alleles for maintaining normal levels of circulating natural IgM antibodies and responses to TI-2 antigens.

## Introduction

Innate-like B cells play significant roles in the early defense against pathogens and, at steady state, anti-inflammatory mediators, such as IL-10, and polyreactive IgM antibodies are secreted by these cell subsets to help maintain homeostasis. There is a clear division of labor between the different innate-like B cells. B-1a cells are thought to secrete most of the natural polyreactive antibodies found in the serum (1). Innate response activator B cells exert protective effects against sepsis by secreting GM-CSF (2). Marginal zone B (MZB) cells, through their location at the interface between the blood and the immune system, help initiate responses against blood-borne antigens [reviewed in Ref. (3)]. B cells regulate inflammation through various mechanisms, including production of anti-inflammatory cytokines, such as IL-10 and IL-35 (4, 5). The main IL-10 producing innate-like B cells, collectively named B10, are found within the MZB (5) and B-1a cell subsets (4, 6). Innate-like B cells are also pivotal for the response against viral and bacterial infections (1). Particularly, the innate-like B cell subsets B-1b and MZB cells are the predominant responding B cells to T-independent type 2 (TI-2) antigens found on the surface of a number of pathogens, such as *Streptococcus pneumoniae*, *Haemophilus influenzae*, and *Neisseria meningitides* (3, 7, 8).

In addition to their distinct roles in homeostasis and response to different stimuli, the innate-like B cell subsets also have distinct developmental pathways. B-1 cells are generated readily from the yolk salk, paraaortic splanchnopleura, and liver during early fetal development (9, 10), while these organs are less effective at generating follicular B cells. In contrast, hematopoietic stem cells from adult bone marrow predominantly generate follicular B and MZB cells (9), collectively referred to as B-2 cells. Immature B cells mature in the spleen and undergo selection at various transitional stages before becoming naive B cells (11). B-2 cells are continuously replenished from the adult bone marrow and diverge into follicular B cells and MZB cells at the transitional B cell stage (12, 13). B-1 cells may develop from a separate progenitor population (14) and mature *via* a phenotypically distinct B-1 transitional B cell intermediate, which is found at high frequencies in the spleen of neonatal mice (15). The different B cell subsets require distinct stimuli for development and maintenance. For example, MZB cells are dependent on Notch signaling, and therefore mice with impaired Notch2 completely lack MZB cells. However, Notch signaling is not required for B-1 or follicular B cell development (12). The distinct B cell subsets also show different requirements for NF-κB signaling (16).

The NF-κB transcription factors, p50 (NF-κB1), p52 (NF-κB2), p65 (RelA), c-Rel (Rel), and RelB, regulate transcription by binding to promoters of target genes. p50 and p52 induce gene transcription by forming heterodimers with p65, c-Rel, or RelB, all of which contain a transactivation domain. In contrast, homodimers of p50 or p52 lack a transactivation domain and thus generally function as repressors of transcription. In classical NF-κB signaling, the NF-κB transcription factors are sequestered in the cytoplasm as dimers of p50:p65 by a protein family known as inhibitors of κB (IκB), including IκB-α, IκB-β, IκB-ϵ, and the p50 precursor p105. The IκB proteins are characterized by their ankyrin repeat structure, which functions to mask nuclear localization signals (17). IκB kinases (IKK), IKK-α (IKK1), IKK-β (IKK2), and IKK-γ (NF-kappa-B essential modulator, NEMO), target IκBs for polyubiquitination and proteasomal degradation, thereby releasing the sequestered NF-κB1 p50 to nuclear localization (18, 19). In lymphocytes, this requires the CARD11, BCL-10, MALT1 (CBM) complex. Through an alternative NF-κB signaling pathway, NF-κB-inducing kinase (NIK) can activate IKK-α, facilitating proteasomal processing of NF-κB2 p100. This ultimately leads to nuclear localization of NF-κB p52/RelB (20). A number of atypical IκB proteins have recently been identified, defined by their ankyrin repeat structure and comprise BCL-3, IκBζ, IκBNS, and IκBη. Atypical IκB proteins may either augment or repress transcription depending on cell type, context, and timing. Recent studies have revealed important roles of atypical IκB proteins in lymphopoiesis and immunological responses [reviewed in Ref. (21)].

Classical NF-κB signaling is required for the generation of B-1 cells, particularly the B-1a subset, which is absent in a number of mouse strains where this pathway has been ablated [reviewed in Ref. (22)]. Reduction in MZB cell numbers is also seen in the absence of classical NF-κB signaling, while follicular B cells are less affected (23, 24). Although relatively little is known about the function of atypical IκB proteins in B cell development, roles for BCL-3 and IκBNS have recently been demonstrated. BCL-3 deficiency leads to increased numbers of MZB cells (25), while decreased B-1 and MZB cellularity was observed upon overexpression of BCL-3 (26). Absence of functional IκBNS leads to reductions in B-1b and MZB cell frequencies (27, 28) and complete absence of B-1a cells, while follicular B cell frequencies are intact (15, 28). In terms of B cell lymphopoiesis, IκBNS-deficient mice thus resemble other mouse strains with impaired classical NF-κB signaling. In addition to the role of classical NF-κB signaling in B cell development, it is also required for normal function of mature B cells. B cells from p50, BCL10, and CARMA1-deficient mice display reduced proliferation and antibody production to anti-IgM, anti-CD40, or LPS compared to wild-type (wt) cells *in vitro* (29–31).

Mice with impaired classical NF-κB signaling have reduced levels of circulating natural IgM and IgG3 antibodies and fail to mount antibody responses to TI-2 antigens *in vivo*. We previously described that mice lacking functional IκBNS due to a mutation in the *nfkbid* gene (*bumble* mice) also display reduced IgM and IgG3 levels and fail to respond to immunization with NP-ficoll (27, 28). Whether the impaired antibody response in mice deficient in classical NF-κB pathway signaling is a consequence of the reduced numbers of B-1 and MZB cells or is due to defects in B cell function remains unknown. Here, we demonstrate that the lack of IκBNS even at the heterozygous state (IκBNS^+^/*bmb*) led to severely reduced antibody responses against TI-2 antigens, suggesting haploinsufficiency for IκBNS in the response to such antigens. Interestingly, unlike homozygous *bumble* mice, the heterozygous mice displayed apparently normal frequencies and numbers of MZB and B-1 cells. This indicates that the reduced responses to TI-2 antigens in these mice are due to a direct requirement of IκBNS in response to B cell receptor engagement rather than a secondary effect due to lack of responding cells.

## Materials and Methods

### Mice

Mice were housed and bred at the animal research facility, MTC, Karolinska Institutet. *Bumble* mice, generated by *N*-ethyl-*N*-nitrosourea (ENU) mutagenesis of C57BL/6J mice, and their wt C57BL/6J counterparts were described previously (28). Animal studies were conducted with Committee for Animal Ethics (Stockholms Norra Djurförsöksetiska nämnd) approval.

### Cell Preparation

Splenocytes were prepared as a single cell suspension using a 70-μm cell strainer. Peritoneal cells were isolated by flushing with cold PBS/1% FBS (5–10 ml). Peritoneal cells were discarded if contaminated with blood. Cell suspensions were diluted in RPMI-1640 supplemented with 2 mM l-glutamine, penicillin (100 IU)–streptomycin (100 μg/ml), and 10% fetal bovine serum (complete RPMI). Splenocyte cell suspensions were washed once in Ca^2+^-free Mg^2+^-free PBS and treated with red blood cell lysis buffer before further processing. B cells were isolated from the spleen using the B cell isolation kit and from the peritoneal cavity using the Pan-B cell isolation kit (Stemcell Technologies). The purity of the isolated populations was ≥90%.

### Immunization

Mice were immunized with 50-μg NP (40)-ficoll (Biosearch Technologies) or a 1:10 dilution of Pneumovax^®^ (Merck & Co.) corresponding to 0.5 μg of each polysaccharide antigen. The antigens were diluted in PBS and 100 μl was injected intraperitoneally (i.p.).

### Adoptive Transfer

Isolated peritoneal B cells (2 × 10^6^ cells) from wt or heterozygous mice were mixed with 50 μg NP (40)-ficoll and injected i.p. into *bumble* mice. Splenic B cells (30 × 10^6^ cells) were injected i.v.

### ELISA

ELISA was performed by coating ELISA plates (Nunc) with polysaccharide antigens: 500 ng/well of NP (25) conjugated with BSA (Biosearch Technologies) or the pneumococcal polysaccharide antigens Type 1 161-X™ or Type 3 169-X™ (both ATCC) or 1:100 dilution of Pneumovax (corresponding to 50 ng/well of each antigen contained in the vaccine). To measure total IgM and IgG3 levels, plates were coated with unconjugated anti-IgM or anti-IgG3 (Southern Biotech). Plates were incubated overnight (4°C). Following washing (PBS + 2% Tween20) and blocking for 1 h with PBS containing 2% dry milk, serum was added in threefold serial dilutions in blocking buffer and incubated for 1.5 h at room temperature (RT) before addition of secondary antibody HRP-coupled anti-IgM or IgG3 (Southern Biotech). The assay was developed with TMB substrate (KPL) followed by 1M H_2_SO_4_ and the OD was read at 450 nm using an Asys Expert 96 ELISA reader (Biochrom).

### ELISpot Assay for Detection of Antibody-Secreting Cells

Detection of total IgM and NP-specific IgM producing cells was performed using enzyme-linked immunosorbent spot (ELISpot) assay. MultiScreen-IP filter plates (Millipore) were pretreated with 70% ethanol and washed in sterile PBS. Plates were coated with 5 μg/ml anti-mouse IgM (Southern Biotech) or 5 μg/ml of NP (25), conjugated with BSA (Biosearch Technologies), diluted in PBS, and incubated overnight at 4°C. The following day, plates were washed in sterile PBS, blocked in complete RPMI medium with 50-μM 2-mercaptoethanol and 10-mM HEPES for 1 h at 37°C, and the indicated cell numbers added in triplicate. Plates were incubated for 17 h at 37°C in 5% CO_2_. Cells were then removed by washing in PBS and 0.1 μg/well of biotinylated anti-mouse IgM (Mabtech) diluted in PBS was added to the wells. After 2 h of incubation at RT, plates were washed and developed with 100 μl of 5-bromo-4-chloro-3-indolyl phosphate/NBT-plus substrate (Mabtech). The reaction was stopped when distinct spots could be observed, by rinsing the plates extensively in tap water. Spots were counted by ELISpot reader (CTL) and analyzed using the Biospot suite (CTL).

### Flow Cytometry

Cells were incubated with Fc block (anti-CD16/32, BD) and stained with fluorochrome-conjugated monoclonal antibodies in PBS/2% FBS using the following antibodies: CD5 Brilliant Violet 421 (S3-7.3), CD19 PE (1D3), CD19 FITC (1D3), CD23 Brilliant Violet 421 (B3B4), CD21 APC (7G6), CD43 APC (S7) (all BD), B220 APC-eFluor 780 (RA3-6B2), CD93 APC (AA4.1) (all eBioscience), and IgM FITC (polyclonal) (Southern Biotech). Data were analyzed in FlowJo v9.6.4 (Treestar).

### Statistics

Differences between groups were analyzed by a Mann–Whitney test (GraphPad Prism v6.0f).

## Results

### Heterozygous *Bumble* Mice Have Reduced Serum IgM Levels and Responses to the TI-2 Antigen NP-Ficoll

**Figure 1:**
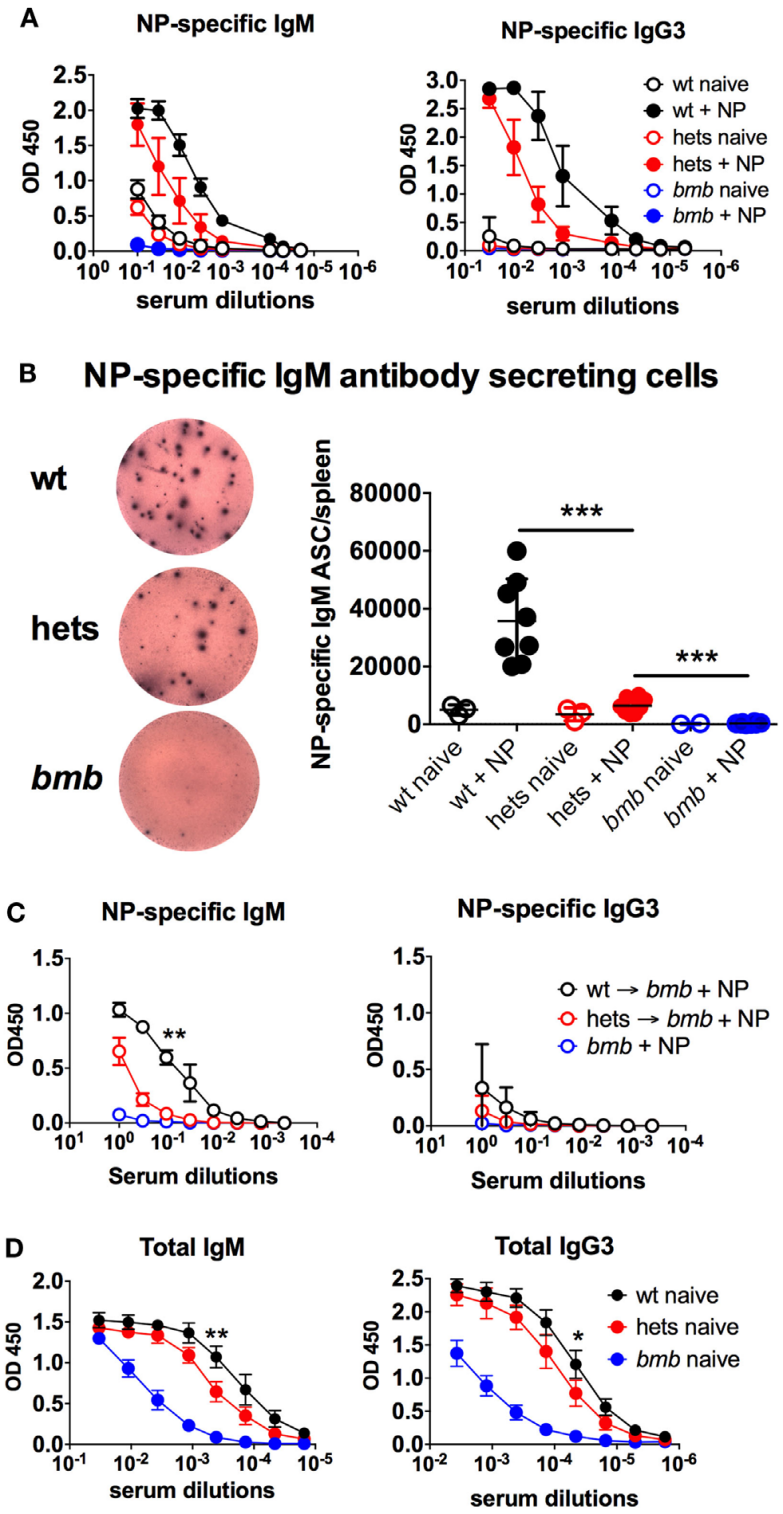
**Heterozygous *bumble* mice have reduced serum IgM levels and responses to TI-2 antigens**. **(A)** Mice were immunized with 50 μg of NP-ficoll intraperitoneally and sacrificed after 6 days. NP-specific serum IgM and IgG3 were determined by ELISA. **(B)** NP-specific IgM antibody-secreting cells were determined by ELISpot. Left panel displays representative wells of wild-type (wt), heterozygous (hets), and *bumble* (*bmb*). **(C)** Isolated peritoneal B cells (2 × 10^6^ cells) from wild-type or heterozygous *bumble* mice were mixed with 50-μg NP (40)-ficoll and injected i.p. into *bumble* mice. The recipient *bumble* mice then received splenic B cells (30 × 10^6^ cells) from wild-type or heterozygous mice injected i.v. NP-specific serum IgM and IgG3 were determined by ELISA 6 days later. **(D)** Total serum IgM and IgG3 were determined by ELISA. Figures represent 8- to 16-week-old aged-matched mice with three to eight per group and results are representative of three **(A,B)** or two **(C)** independent experiments. Graphs display mean + SD. Statistically significant differences between *bumble*, *bumble* heterozygotes, and wt mice are indicated by *p < 0.05, **p < 0.01, and ***p < 0.001 by Mann–Whitney test.

In an ENU mutagenesis screen for antibody response defects, we identified a role of IκBNS for B cell development and function. We observed that mice homozygous for a specific mutation in *nfkbid* lacked responses to the TI-2 antigen NP-ficoll and had reduced levels of circulating IgM and IgG3 antibodies (28). These mice, named *bumble*, were found to have a T → G transversion in the conserved donor splice site in the fourth intron of *nfkbid*, the gene encoding IκBNS. This mutation is predicted to prevent splicing of the fourth intron from the *nfkbid* transcript, resulting in a premature stop codon after exon 4. Such a transcript would likely be targeted for nonsense-mediated decay or encode only 65 of 327 amino acids (aa) encoded by full-length *nfkbid*, and therefore not be expected to retain any function. The phenotype for *bumble* mice was copied in mice with a targeted mutation in the *nfkbid* gene (28) and was similar in IκBNS knockout mice (27). Here, we report that mice heterozygous for the *bumble* mutation displayed reduced NP-specific IgM and IgG3 antibody responses after NP-ficoll immunization (Figure 1A). Furthermore, when analyzing isolated splenocytes for NP-specific IgM antibody-secreting cells (ASC), we observed significantly (*p* < 0.001) lower numbers in *bumble* heterozygous compared to wt mice (Figure 1B). To test if the TI-2 antigen response defect in heterozygous *bumble* mice was due to a B cell intrinsic defect, we transferred isolated splenic and peritoneal B cells to *bumble* mice and immunized them with NP-ficoll. *Bumble* mice that had not received isolated B cells did not respond to immunization, whereas *bumble* mice that had received wt B cells did. *Bumble* mice that had received B cells from heterozygous *bumble* mice had an intermediate response to NP-ficoll immunization, suggesting that the defective antibody response to TI-2 antigens in heterozygous *bumble* mice is due to a B cell intrinsic defect (Figure 1C). Similarly to *bumble* homozygotes, mice with the heterozygous *bumble* mutation displayed significantly reduced total serum levels of IgM and IgG3 antibodies (*p* < 0.05) (Figure 1D). Thus, relative to homozygous *bumble* mice and wt mice, heterozygous *bumble* mice presented with an intermediary phenotype in terms of response to NP-ficoll and levels of circulating IgM and IgG3 antibodies.

### Fewer IgM Antibody-Secreting Cells in Heterozygous *Bumble* Mice

**Figure 2:**
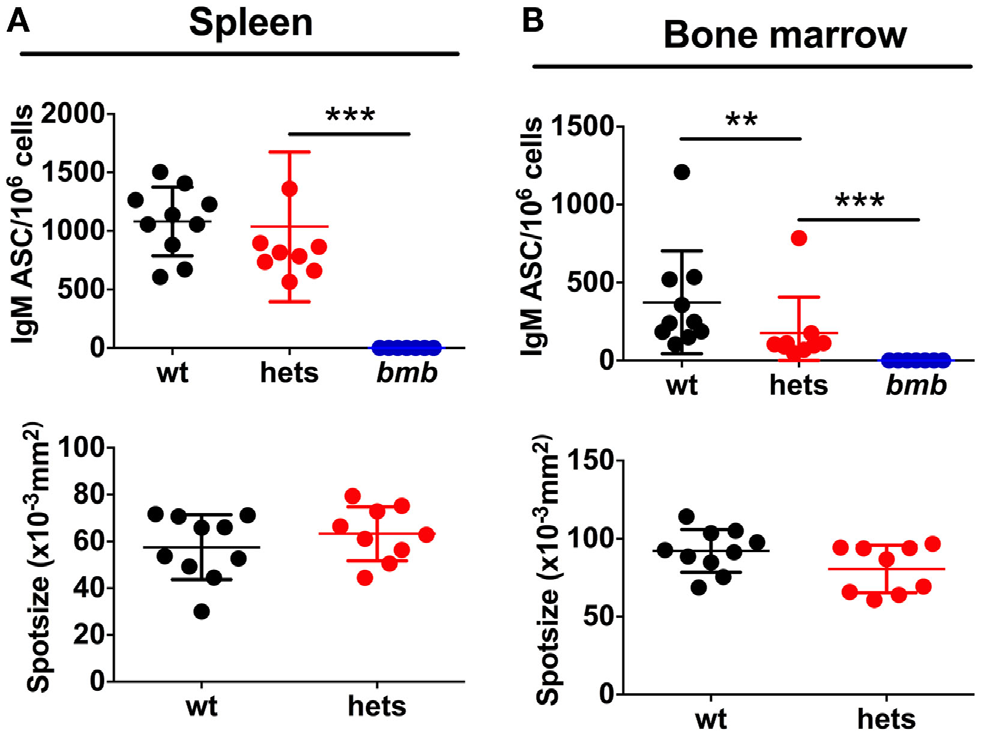
**Heterozygous *bumble* mice have fewer IgM-secreting B cells**. Cell suspensions in serial dilutions were plated on ELISpot plates coated with anti-IgM. IgM antibody-secreting cells (ASC) in **(A)** spleen and **(B)** bone marrow. Upper panels display IgM ASCs per 10^6^ cells and lower panels indicate mean spot size. Spot size is influenced by the number of spots per well. Therefore, wells containing similar numbers of spots across groups were used to calculate spot size (range 10–30 spots for bone marrow and 30–70 for spleen). Data were compiled from two experiments.

We next asked if the reduced IgM levels in heterozygous *bumble* mice were due to fewer IgM-secreting B cells or if less IgM was produced per cell. To assess this, we performed ELISpot for total IgM producing splenic and bone marrow B cells. The same numbers of cells were plated from wt, heterozygous, and homozygous *bumble* mice. As expected, no IgM ASCs were detected from homozygous *bumble* mice. The fraction of splenic B cells spontaneously secreting IgM in heterozygous *bumble* mice was slightly lower than in wt mice, although this difference was not statistically significant (*p* = 0.23) (Figure 2A). The frequency of IgM ASCs in bone marrow was significantly lower in heterozygous *bumble* mice than in wt mice (*p* < 0.01). As an indication of the amount of IgM produced per IgM ASC, we evaluated the mean spot size. We observed no measurable differences in mean spot size between heterozygous and wt mice (Figure 2B). These data indicate that the reduced levels of natural IgM antibodies in the serum is due to fewer IgM ASCs in the heterozygous *bumble* rather than less IgM secreted per ASC.

### Heterozygous *Bumble* Mice Have Normal Frequencies of the Major B Cell Subsets

**Figure 3:**
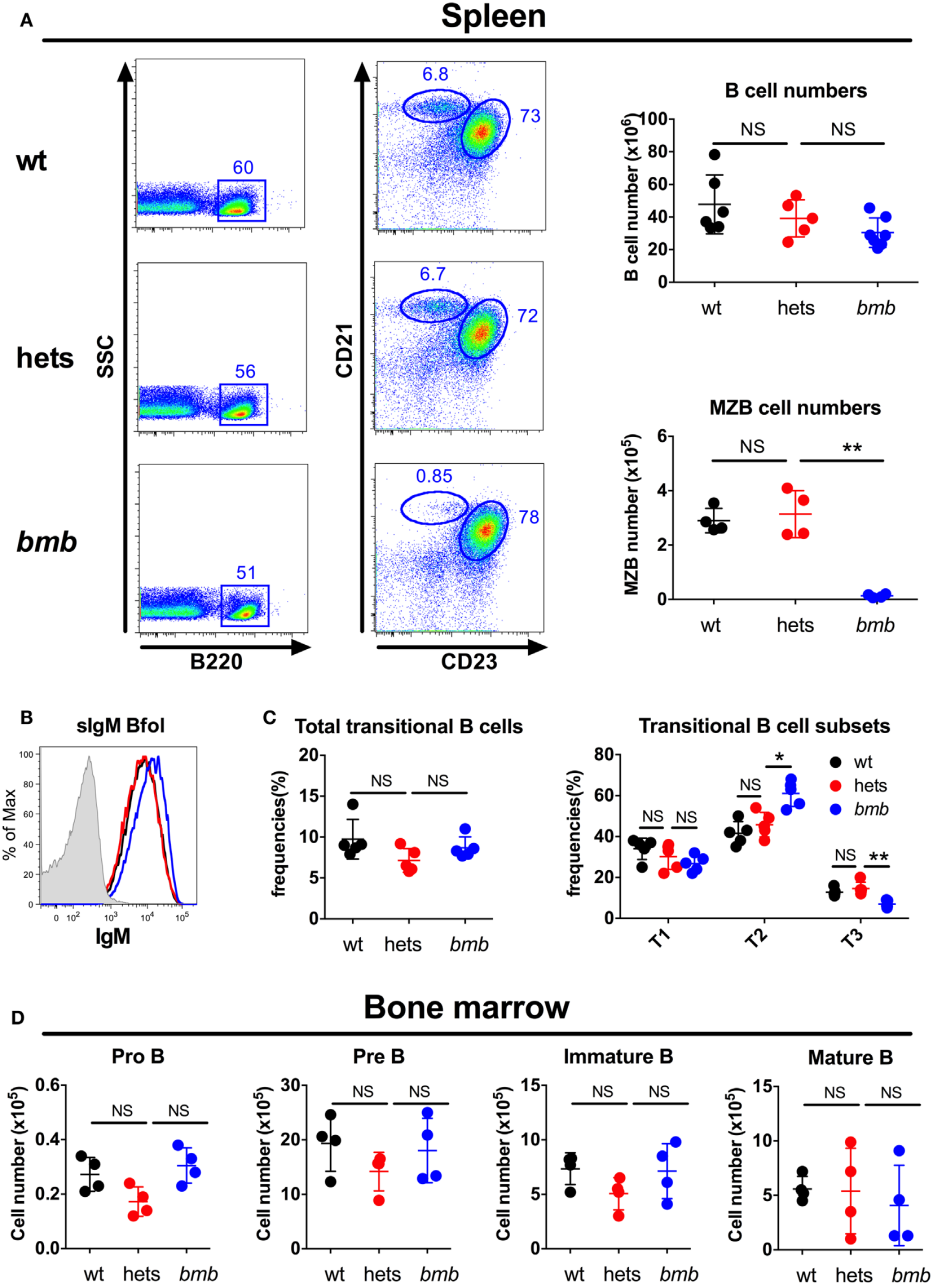
**Heterozygous *bumble* mice have normal frequencies of the major B cell subsets**. Total B cells, marginal zone B cells (MZB), and transitional B cells were stained by FACS in wild-type (wt), heterozygous (hets), and *bumble* (bmb) mice. **(A)** Left panel: representative staining of marginal zone B cells (MZB, B220^+^, CD23^−^, and CD21^hi^) and follicular B cells (B220^+^, CD23^+^, and CD21^+^). Right panel: total B cell (B220^+^) and MZB cell numbers. **(B)** Representative staining of surface IgM (sIgM) on wt (black), heterozygous (blue), and *bumble* (blue) follicular B cells. **(C)** Splenic transitional B cells (B220^+^ and CD93^+^), further subdivided into T1 (CD23^−^IgM^+^), T2 (CD23^+^IgM^+^), and T3 (CD23^+^IgM^lo^). **(D)** Bone marrow B cell progenitors, pro B (B220^+^CD43^+^CD19^+^CD93^+^), pre B (B220^+^CD43^−^IgM^−^CD93^+^), immature B (B220^+^CD43^−^IgM^+^CD93^+^), and mature B cells (B220^+^CD43^−^IgM^+^CD93^−^). For transitional and progenitor B cell gating strategy, see Figure S1 in Supplementary Material. Figures represent 8- to 16-week-old mice with four to seven mice per group, and results are representative of at least two independent experiments. Graphs display mean + SD. Statistically significant differences between *bumble*, *bumble* heterozygotes, and wt mice are indicated by *p < 0.05 by Mann–Whitney test.

**Figure 4:**
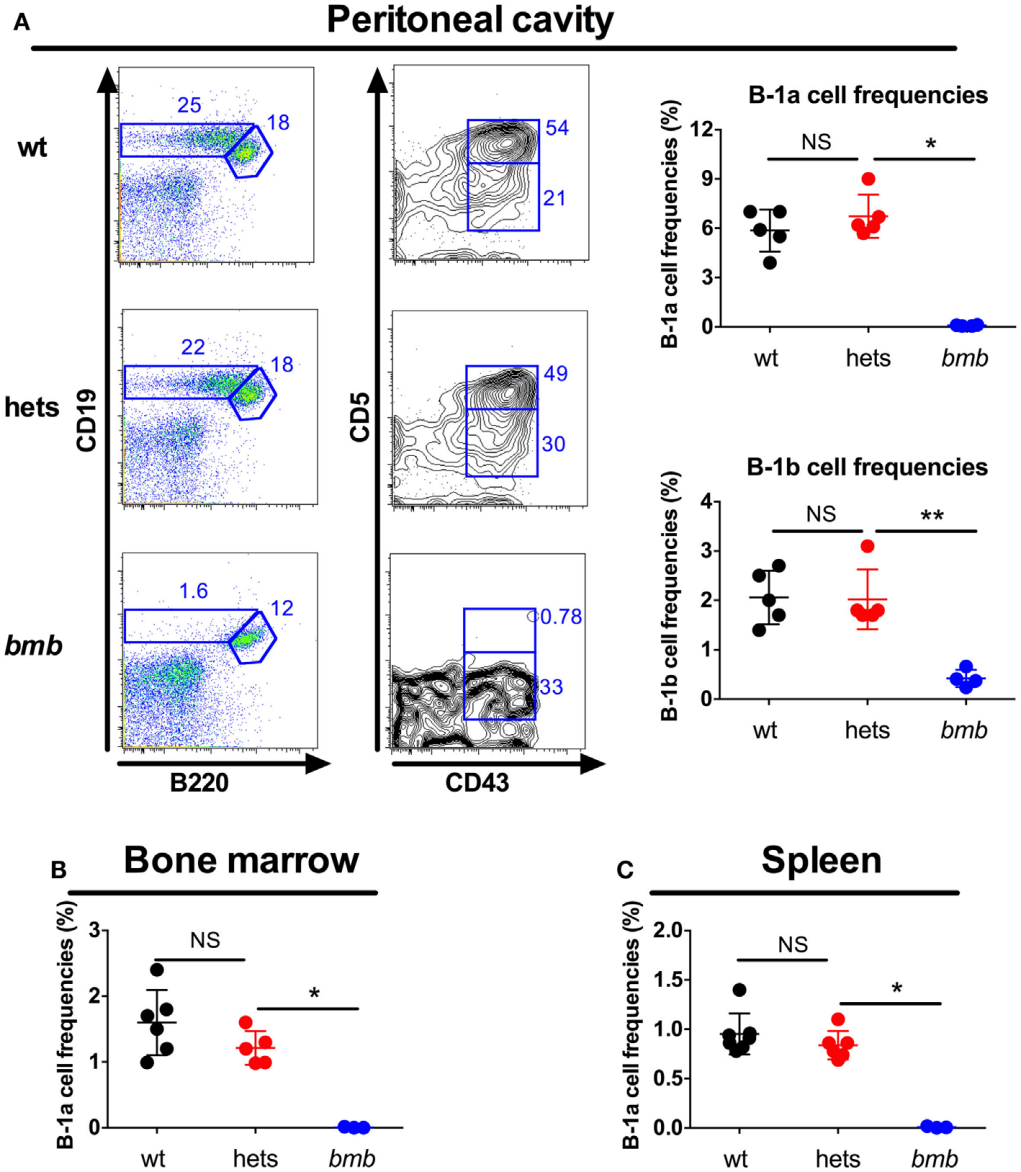
**Heterozygous *bumble* mice have normal frequencies of B-1 cells**. Wild-type (wt), heterozygous *bumble* (hets), and *bumble* (*bmb*) cells were stained for B-1 cells. **(A)** Peritoneal B-1 cells identified as CD19^hi^B220^lo^CD43^+^ (B1b) and CD19^hi^B220^lo^CD43^+^CD5^+^ (B1a). B-1a cells were identified as CD93-IgM^+^CD19^hi^B220^lo^CD5^+^ in **(B)** bone marrow and **(C)** spleen. Representative stainings of B-1a cells in bone marrow and spleen are shown in Figure S2 in Supplementary Material. Figures represent 8- to 16-week-old mice with four to seven mice per group, and results are representative of at least two independent experiments. Graphs display mean + SD. Statistically significant differences between *bumble*, *bumble* heterozygotes, and wt mice are indicated by *p < 0.05 and **p < 0.01 by Mann–Whitney test.

The main responding B cell subsets against TI-2 antigens are B-1b cells and MZB cells (3, 7, 8), while B-1a cells are believed to produce most of the natural IgM antibodies found in the serum at steady state (1). Homozygous *bumble* mice had normal numbers of follicular B cells, but completely lacked B-1a cells, and displayed a severe reduction in the frequencies of B-1b and MZB cells (15, 28). Transfer of wt peritoneal cells to *bumble* mice completely restored serum natural IgM levels and partly restored the antibody response to immunization with NP-ficoll (15). This suggested that the lack of B-1b cells in homozygous *bumble* mice formed the basis for the impaired response to T-independent antigens. To investigate if heterozygous *bumble* mice have defects in B cell development, we used the following strategy to phenotypically distinguish the major B cell subsets. B220 is expressed by all B-2 cells (MZB and follicular B cells), but at lower levels on most B-1 cells. CD11b and CD43 are expressed by B-1 cells, and CD5 is expressed by the B-1a cell subset. CD23 is expressed by B-2 cells, but not MZB cells, while MZB cells express high levels of CD21. When analyzing the different B cell subsets in heterozygous *bumble* mice, we found that neither overall splenic B cell nor MZB cell numbers were significantly different between wt mice and heterozygous *bumble* mice. In contrast, mice homozygous for the *bumble* mutation had significantly reduced MZB cell numbers as expected (*p* < 0.01) (Figure 3A). The increased surface IgM level seen in homozygous *bumble* mice (28) was not observed in the heterozygous state (Figure 3B). Furthermore, homozygous *bumble* mice have decreased frequencies of transitional T3 B cells (15), while in heterozygous mice, the T3 B cell frequencies were similar to those of wt mice (Figure 3C). No differences in bone marrow B cell progenitor populations were observed between wt, homozygous, and heterozygous *bumble* mice (Figure 3D). As we reported previously, homozygous *bumble* mice completely lacked B-1a cells and had significantly reduced B-1b cell frequencies (*p* < 0.01) (15). In contrast, peritoneal B-1a and B-1b cell frequencies were similar in wt and heterozygous *bumble* mice (Figure 4A). Similarly, bone marrow B-1a cell and spleen B-1a cell frequencies were indifferent between heterozygous *bumble* and wt mice (Figures 4B,C). Overall, these data indicate that the development and maintenance of all the major B cell subsets occur normally in heterozygous *bumble* mice.

### Impaired Antibody Response to the TI-2 Vaccine Pneumovax in Heterozygous *Bumble* Mice

**Figure 5:**
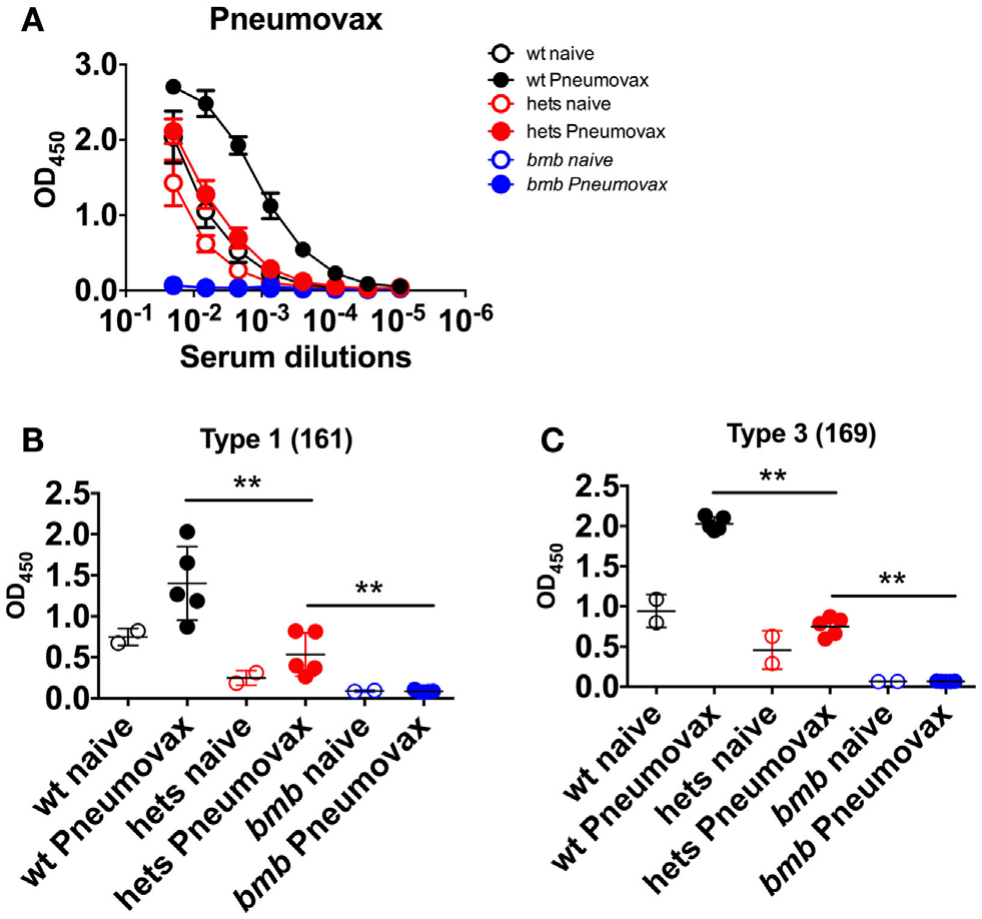
**Heterozygous *bumble* mice have reduced antibody responses to immunization with pneumococcal polysaccharide vaccine**. Wild-type (wt), heterozygous *bumble* (hets), and *bumble* (bmb) mice were immunized with Pneumovax pneumococcal polysaccharide vaccine and sacrificed after 6 days. Serum antibody responses were analyzed by ELISA against **(A)** Pneumovax, **(B)** type 1 polysaccharide antigen 161-X, and **(C)** type 3 polysaccharide antigen 169-X. Figures represent 14- to 16-week-old mice with five mice per immunized group, and results are representative of two independent experiments. Graphs display mean + SD. Statistically significant differences between *bumble*, *bumble* heterozygotes, and wt mice are indicated by **p < 0.01 by Mann–Whitney test.

We next investigated if the impaired response to TI-2 antigens in heterozygous *bumble* mice would extend to the clinically relevant human polysaccharide vaccine Pneumovax. To this end, wt, heterozygous, and homozygous *bumble* mice were immunized with Pneumovax intraperitoneally. By 6 days post-immunization, wt mice mounted a strong antibody response against the Pneumovax vaccine antigens. No response was observed in homozygous *bumble* mice, while heterozygous *bumble* mice displayed an intermediary response (Figure 5A). We also investigated the Pneumovax-elicited responses to the polysaccharide antigens type 1 161-X (Figure 5B) and type 3 169-X (Figure 5C). Similarly to total Pneumovax-specific antibodies, the responses against both polysaccharides were significantly diminished in heterozygous *bumble* mice compared to in wt mice (*p* < 0.01).

## Discussion

The data presented here demonstrate that a heterozygous mutation in IκBNS causes a significant impairment of B cell function. Mice that express one wt allele and one allele containing the *bumble* mutation in *nfkbid* had diminished antibody responses to T-independent type II antigens and reduced levels of circulating natural IgM and IgG3 antibodies. The *bumble* mutation causes a premature stop codon after exon 4 in the *nfkbid* gene and the resulting transcript encodes only 65 of the 327 aa of the full-length IκBNS (28). The resulting mRNA transcript is likely targeted for degradation by nonsense-mediated decay. Thus, the spontaneous secretion of natural antibodies and responses to TI-2 antigens in *bumble* heterozygotes are likely impaired as a result of haploinsufficiency for the *nfkbid* gene.

Previous work has shown that p50^−/−^, BCL10^−/−^, and IκBα hypermorphic mice fail to respond to immunization with TI-2 antigens (32, 33), illustrating that this defect is a general feature of deficiency in the classical NF-κB signaling pathway. Similar to other proteins of the classical NF-κB pathway, IκBNS also plays an important role for maintaining normal levels of serum natural antibodies and for the antibody response against TI-2 antigens (27, 28). Notably, the major B cell subsets responsible for natural antibody production and response to TI-2 antigens, B-1 and MZB cells are lacking in mice with impaired classical NF-κB signaling, which is also similar for mice with non-functional IκBNS (15, 27, 28). It was therefore interesting that heterozygous *bumble* mice had decreased serum IgM and IgG3 levels and defective responses to TI-2 antigens, despite having apparently normal numbers and frequencies of B-1 and MZB cells. This suggested that the lack of specific B cell subsets is not the sole reason for the reduced levels of natural antibodies and response to TI-2 antigens in IκBNS-deficient mice. Rather, this indicated a more direct role of IκBNS for these aspects of immunity downstream of B cell receptor signaling. Particularly, the lack of response to TI-2 antigens of heterozygous *bumble* mice may be due to a B cell intrinsic defect in activation or ASC differentiation, although it is also possible that the BCR repertoire may be altered when IκBNS is only expressed from one allele. We did not observe any reduction in B-1a, MZB, or follicular B cell numbers in heterozygous *bumble* mice, suggesting that one functioning allele of *nfkbid* is enough to facilitate normal B cell development. However, we cannot rule out the possibility that a modest impairment of B cell development is masked by homeostatic proliferative mechanisms or altered bone marrow output to control distribution of immune cells (34).

There are only few reports of heterozygous mutations in the NF-κB pathways leading to loss of B cell function. Hypermorphic heterozygous mutations in IκBα cause impaired antibody responses (33) and CARMA1-deficient mice lacking CARD (ΔCARD) showed defective B cell proliferation at the heterozygous state (35). Since ΔCARD was found to act as a dominant-negative inhibitor of TCR-induced NF-κB activation, the impaired B cell function in heterozygous ΔCARD-deficient mice may be due to the truncated CARMA1 protein interfering with function of the wt protein (36). Protein kinase C-β (PKCβ) initiates a phosphorylation cascade that activates the CBM complex downstream BCR signaling. Heterozygous missense mutations in the gene encoding PKCβ were found to lead to impaired antipolysaccharide antibody responses and reduced natural antibody levels, despite apparently normal B cell development (37).

Several clinical cases with mutations in NF-κB proteins have demonstrated important roles of both the classical and alternative NF-κB signaling pathways for B cell development and function in humans. Hypermorphic mutations in the gene encoding IκBα lead to impaired phosphorylation-driven degradation of the mutant protein and thereby reduced NF-κB signaling (38). Hypermorphic heterozygous mutations in IκBα cause ectodermal dysplasia with immunodeficiency as evidenced by recurrent severe infections (38, 39). These patients have increased numbers of B and T cells, but display both B and T cell functional defects (39). Notably, the patient symptoms and lymphocyte functional defects could to a large extent be reproduced when introducing one of the hypermorphic IκBα mutations (S32I) to mice (33). Similar symptoms to IκBα hypermorphs are evident in patients with NEMO deficiency and B cells from these patients do not respond to CD40 ligation (40). More recently, several patients with mutations in the CBM complex have been described. Combined immunodeficiency (CID) resulting from impaired classical NF-κB signaling due to CARMA1 deficiency was associated with hypogammaglobulinaemia, impaired BAFF-R expression and a block of B cell maturation at the transitional B cell stage (41, 42). Human MALT1 deficiency, also manifested by CID, was associated with lack of MZB cells and failure to respond to Pneumovax vaccination (43, 44), a phenotype that is very similar to that seen in the corresponding mouse model (45). Rapid advances in identifying genes underlying human immunodeficiencies will reveal if also IκBNS plays a role in this disease group.

So far, there are few reports of heterozygous mutations in the NF-κB pathway leading to immunodeficiency in humans. Heterozygous gain-of-function mutations in the gene encoding CARMA1 were shown to result in constitutive NF-κB activity and are manifested by lymphocytosis but impaired memory B cells and low TI-2 responses (46, 47). It is interesting to speculate if heterozygous mutations in genes encoding components of BCR signaling, including classical NF-κB pathway mediators, may contribute to the observed variability in antipolysaccharide immune responses in the human population (48, 49). We describe here that a heterozygous mutation in the *nfkbid* gene encoding the atypical IκB protein IκBNS led to reduced steady state IgM and IgG3 antibody levels and impaired response to vaccination with TI-2 antigens in mice. Heterozygous mutations in genes of the NF-κB pathway could potentially lead to haploinsufficiency for B cell function, resulting in lower antibody responses to vaccination and increased susceptibility to infection, which should be considered in future studies.

## Author Contributions

GKP designed and performed the experiments and wrote the manuscript. MÁ, JS, and SK performed the experiments. CA and BB designed the experiments. GKH designed the experiments and wrote the manuscript. All authors have approved the manuscript.

## Conflict of Interest Statement

The authors declare that the research was conducted in the absence of any commercial or financial relationships that could be construed as a potential conflict of interest.

## Supplementary Material

Figure S1 **Gating strategy for identifying transitional and progenitor B cells**. **(A)** Representative staining of wild type splenocytes for transitional B cells (CD93^+^B220^+^) and the transitional B cell populations gated as indicated. **(B)** Representative staining of wild type bone marrow for progenitor and mature B cell populations. Pro B (B220^+^CD43^+^CD19^+^CD93^+^), pre B (B220^+^CD43^-^IgM^-^CD93^+^), immature B (B220^+^CD43^-^IgM^+^CD93^+^), and mature B cells (B220^+^CD43^-^IgM^+^CD93^-^).

Figure S2 **Representative stainings of bone marrow and spleen B-1a cells. B-1a cells were identified as CD93-IgM+CD19hiB220loCD5+ in (A) bone marrow and (B) spleen**.
